# A Psychometric Evaluation of the Dysphagia Handicap Index Using Rasch Analysis

**DOI:** 10.3390/jcm13082331

**Published:** 2024-04-17

**Authors:** Reinie Cordier, Annette Veronica Joosten, Bas J. Heijnen, Renée Speyer

**Affiliations:** 1 Department of Social Work, Education and Community Wellbeing, Northumbria University, Newcastle upon Tyne NE1 8ST, UK; reinie.cordier@northumbria.ac.uk; 2 Curtin School of Allied Health, Curtin University, Perth, WA 6102, Australia; 3 Department of Health & Rehabilitation Sciences, Faculty of Health Sciences, University of Cape Town, Cape Town 7700, South Africa; 4 School of Allied Health, Australian Catholic University, Melbourne, VIC 3065, Australia; annette.joosten@acu.edu.au; 5 Department of Otorhinolaryngology and Head and Neck Surgery, Leiden University Medical Centre, 2333 ZA Leiden, The Netherlands; b.j.heijnen@lumc.nl; 6 Department Special Needs Education, University of Oslo, NO-0371 Oslo, Norway; 7 MILO Foundation, Centre for Augmentative and Alternative Communication, 5482 JH Schijndel, The Netherlands

**Keywords:** psychometrics, measurement, Rasch analysis, item response theory, swallowing disorders, deglutition, oropharyngeal dysphagia

## Abstract

**Background/Objectives**: The Dysphagia Handicap Index (DHI) is commonly used in oropharyngeal dysphagia (OD) research as a self-report measure of functional health status and health-related quality of life. The DHI was developed and validated using classic test theory. The aim of this study was to use item response theory (Rasch analysis) to evaluate the psychometric properties of the DHI. **Methods**: Prospective, consecutive patient data were collected at dysphagia or otorhinolaryngology clinics. The sample included 256 adults (53.1% male; mean age 65.2) at risk of OD. The measure’s response scale, person and item fit characteristics, differential item functioning, and dimensionality were evaluated. **Results**: The rating scale was ordered but showed a potential gap in the rating category labels for the overall measure. The overall person (0.91) and item (0.97) reliability was excellent. The overall measure reliably separated persons into at least three distinct groups (person separation index = 3.23) based on swallowing abilities, but the subscales showed inadequate separation. All infit mean squares were in the acceptable range except for the underfitting for item 22 (F). More misfitting was evident in the Z-Standard statistics. Differential item functioning results indicated good performance at an item level for the overall measure; however, contrary to expectation, an OD diagnosis presented only with marginal DIF. The dimensionality of the DHI showed two dimensions in contrast to the three dimensions suggested by the original authors. **Conclusions**: The DHI failed to reproduce the original three subscales. Caution is needed using the DHI subscales; only the DHI total score should be used. A redevelopment of the DHI is needed; however, given the complexities involved in addressing these issues, the development of a new measure that ensures good content validity may be preferred.

## 1. Introduction

Oropharyngeal dysphagia (OD) or deglutition disorders are associated with dehydration, malnutrition, aspiration pneumonia, and even death [1,2,3,4]. Apart from affecting physical well-being, dysphagia has a major impact on a person’s quality of life [5,6]. Therefore, self-report measures are important for dysphagia assessment [7,8].

Patient self-evaluation comprises two different aspects: functional health status (FHS) and health-related quality of life (HR-QoL) [4,8]. FHS is the impact of a given disease on the ability to perform tasks in multiple domains (including physical, social, role, and psychological functioning). The FHS aims to quantify the symptomatic severity and (loss of) function due to the disease and/or treatment and the effects on daily life as experienced by the individual at a particular point in time [7]. HR-QoL refers to the unique personal perception of someone’s health, considering social, functional, and psychological issues [9]. Although considered two distinct concepts, self-evaluation questionnaires frequently combine both FHS and HR-QoL, without distinguishing disease-related functioning from disease-related quality of life as experienced by the patient [10].

A measure’s robust psychometric properties must be demonstrated before implementing it in healthcare or research [11,12]. Two systematic reviews summarising the evidence for the measurement properties of patient self-reported measures developed for people with dysphagia reported poor and incomplete psychometric data [13,14]. Nearly all studies apply the principles of Classic Testing Theory (CTT) when evaluating the psychometric robustness of measures, while only a few studies use the more contemporary item response theory (IRT) framework [15,16,17]. CTT and IRT are the most common frameworks used in instrument development and the evaluation of psychometric properties [18]. CTT analyses evaluate the performance of a measure as a whole, whereas the IRT framework uses the item as the unit of analysis. Also, contrary to CTT, results in IRT are not bound by the test population [11,19]. Consequently, psychometric studies using CTT analyses may yield different results than studies incorporating IRT principles and, therefore, may lead to different recommendations or guidelines about which measures to implement in clinics or research [10,15,20].

Commonly used self-report measures for patients with dysphagia include the MD Anderson Dysphagia Inventory (MDADI; [21]), the Swallowing Quality of Life Questionnaire (SWAL-QOL; [22]), the Eating Assessment Tool (EAT-10; [23]) and the Dysphagia Handicap Index (DHI; [24]). To date, only two self-reported measures targeting people with oropharyngeal dysphagia have been evaluated using IRT analyses: the SWAL-QOL [15] and the EAT-10 [10,20,25]. In contrast to previous studies reporting on both measures’ good validity and reliability using CTT, more recent studies using IRT analyses identified major psychometric weaknesses in both measures, calling for further evaluation of the underlying structure and possible redevelopment using IRT.

Overall, both CTT and IRT principles should be considered when developing new instruments and evaluating the psychometric properties of existing measures. Repeating limited CTT analyses for a single measure (repeated cross-cultural validation of a measure into numerous languages; for example, [26]) may not further strengthen the psychometric evidence to support its use, while introducing the IRT framework alongside CTT principles will lead to a better understanding of the robustness of the psychometric properties of a measure, prioritising the quality over quantity of psychometric analyses.

Originally, the Dysphagia Handicap Index (DHI) was developed and validated by Silbergleit, Schultz [24] using CTT. The DHI is a patient-administered questionnaire comprising 25 items across three subscales: the emotional (7 items), functional (9 items), and physical aspects of individuals’ lives (9 items) [24]. Items are scored using a three-point ordinal scale (i.e., never = 0; sometimes = 2; or always = 4), with higher scores indicating a higher degree of disability or impact on patients’ quality of life. The questionnaire concludes with a single question on the patients’ perceived severity of dysphagia using a seven-point scale with three anchor values (1 = normal swallowing; 4 = moderate swallowing problem; and 7 = severe swallowing problem). The item descriptions are provided in Table 1.

After its publication, several psychometric studies have been conducted on the DHI to determine its psychometric properties using CTT analyses; none have used IRT principles. For example, many studies evaluated hypothesis testing (e.g., convergent validity; [27,28]) and cross-cultural validity (e.g., [29,30]), while minimal data on responsiveness can be obtained from the literature, and no data on measurement error and structural validity have been published.

To address the gap in research, this study aimed to apply an IRT approach to determine the psychometric robustness of the DHI. Using the Rasch measurement model, this study evaluated the response scale, the person and item fit characteristics, differential item functioning, and the scale’s dimensionality.

**Table 1:** Dysphagia Handicap Index items and domains.

Item #	Domain	Item Description
1	1P	I cough when I drink liquids.
2	2P	I cough when I eat solid food.
3	3P	My mouth is dry.
4	4P	I need to drink fluids to wash food down.
5	5P	I’ve lost weight because of my swallowing problem.
6	1F	I avoid some foods because of my swallowing problem.
7	2F	I have changed the way I swallow to make it easier to eat.
8	1E	I am embarrassed to eat in public.
9	3F	It takes me longer to eat a meal than it used to.
10	4F	I eat smaller meals more often due to my swallowing problem.
11	6P	I have to swallow again before food will go down.
12	2E	I feel depressed because I cannot eat what I want.
13	3E	I do not enjoy eating as much as I used to.
14	5F	I do not socialise as much due to my swallowing problem.
15	6F	I avoid eating because of my swallowing problem.
16	7F	I eat less because of my swallowing problem.
17	4E	I am nervous because of my swallowing problem.
18	5E	I feel handicapped because of my swallowing problem.
19	6E	I get angry at myself because of my swallowing problem.
20	7P	I choke when I take my medication.
21	7E	I am afraid that I will choke and stop breathing because of my swallowing problem.
22	8F	I must eat another way (e.g., feeding tube) because of my swallowing problem.
23	9F	I’ve changed my diet due to my swallowing problem.
24	8P	I feel a strangling sensation when I swallow.
25	9P	I cough up food after I swallow.

*Notes*. Item description from Silbergleit, Schultz [28]; Blue = physical items; green = functional items; pink = emotional items.

## 2. Methods

### 2.1. Participants and Procedure

Prospective, consecutive patient data were collected from January 2017 to February 2018 at clinics for dysphagia or otorhinolaryngology at the Leiden University Medical Center, the Netherlands. Only adult patients (i.e., 18 years and older) at risk of dysphagia and who underwent either a videofluoroscopic swallowing study (VFSS) or a fiberoptic evaluation of swallowing (FEES) were included in this study. Patients with severe cognitive problems or esophageal dysphagia were excluded.

All patients completed the DHI independently, after which a VFSS or FEES was performed as part of standard clinical care. The diagnosis of OD was confirmed through a visuoperceptual evaluation of VFSS or FEES recordings by an experienced speech and language pathologist and/or laryngologist. Further, patient characteristics were collected on both age and gender, in addition to oral intake data (i.e., Fois Oral Intake Scale [FOIS]) [31] as completed by the speech and language pathologist.

In line with COSMIN criteria for adequate sample size for psychometric studies [32], the sample size needed to be five times the number of items, with a minimum sample size of 100. This study was approved by the local Medical Ethics Committee Leiden (approval code: G16.100; date: 17 January 2017) at the Leiden University Medical Center.

### 2.2. Instrument

In 2012, a prototype patient self-report DHI was developed based on a composite series of 60 complaints from dysphagia patients over a one-month period [24]. Twenty-one items were eliminated (i.e., item total correlations *r* < 0.50 [*n* = 21] or redundancy/similar wording [*n* = 14]). Four items with low item total correlations were included in the final DHI as they were considered by the authors to have high content validity or provide pertinent clinical information. The final DHI version was subsequently reduced to 25 items across three subscales: an emotional (7 items), a functional (9 items), and a physical subscale (9 items). The authors chose three response levels to facilitate patients’ understanding of response requirements and added a final item on dysphagia severity as perceived by the patient.

### 2.3. Statistical Analysis

**Figure 1:**
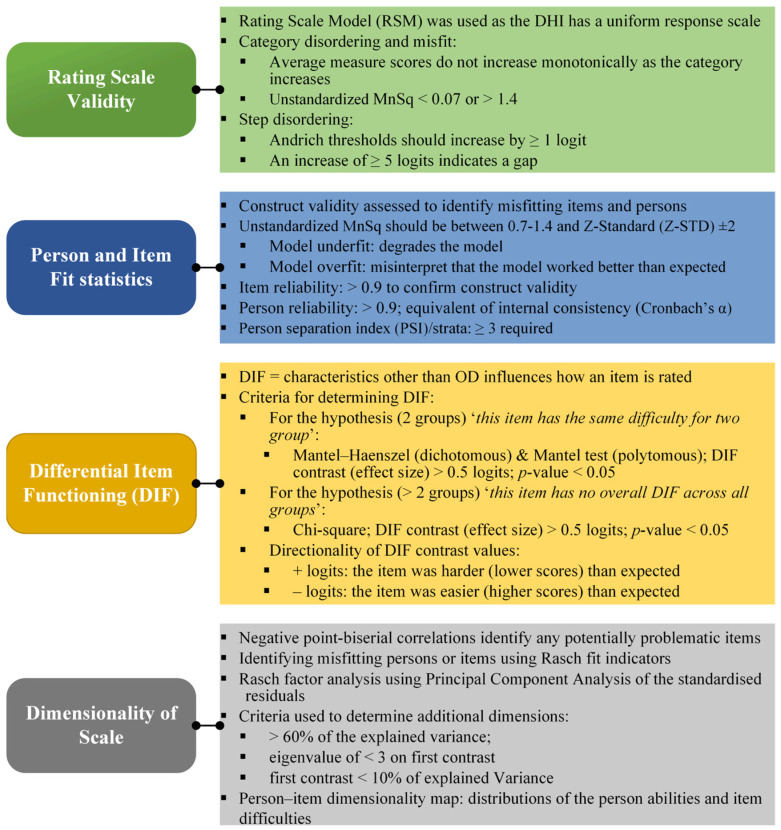
Rasch analysis (item response theory): domains being evaluated.

Rasch analyses were employed to evaluate the reliability and validity of the DHI. Winsteps version 3.92.0 [33] was used to analyse the data, using the joint maximum likelihood estimation rating scale estimation [34]. The initial steps were to analyse all 25 DHI items. An iterative process was then used to remove poor-fitting items in various combinations and re-run the analysis to obtain the best overall item fit, person separation, and dimensionality statistics. All investigations included the analyses as described below. Figure 1 provides a schematic representation of all the Rasch domains that were evaluated.

### 2.4. Rating Scale Validity

To confirm whether the ordinal response scale for all items stays true to the assumption that higher ratings indicate “more” and lower ratings indicate “less” within the DHI measure, a Rating Scale Model (RSM) was used to examine the rating scale validity. The three situations in which the partial credit model in Winsteps can be used [34] do not apply to the DHI scale structure, and all DHI items have the same scale structure. To align with the DHI response options, the original categories (i.e., never = 0; sometimes = 2; or always = 4) were recoded as Never (0), Sometimes (1), and Always (2) to comply with Rasch requirements for an ordinal scale [19].

Category response data were examined for an even distribution or category disorder to determine if the rating response scales were being used in an expected manner. Non-uniformity or category disordering may occur when poorly designed items that do not measure the construct are included. Average measure scores that increase monotonically as the category increases indicate ordered categories. Misfitting categories and disordering, indicated by mean squares (MnSq) outside 0.7–1.4, can be considered for collapsing into an adjacent category [19].

To assess step disordering, Andrich thresholds were used to estimate the equal probability of response in either of the two adjacent categories. Andrich thresholds measure the distance between categories, and it is expected that such distance progresses monotonically, without overlap or with too large a gap. Where step disordering is identified, the category may define a narrow section of the variable, but step disordering does not imply that the category definitions are out of sequence. On a 5-category scale, an increase of at least 1.0 logit indicates distinct categories within the measure. An increase of >5.0 logits indicates gaps in the variable [35].

### 2.5. Person and Item Fit Statistics

Fit statistics, reported as log odd units (logits), were used to assess construct validity. Patterns of responses for each person and misfitting items were analysed to determine the reliability of an individual’s responses. Logits also indicate whether the items contribute to the main construct (i.e., swallowing difficulty). Infit and outfit are both described as unstandardised MnSq or Z-Standard (Z-STD) statistics. Infit and outfit MnSqs should be close to 1.0 with an acceptable range of 0.7–1.4 [36]. Infit and outfit Z-STD statistics should be close to 0 with an acceptable range of ±2 [36]. Where underfitting is found, further investigation is required to understand the reason. Though underfitting degrades the model, the same is not always true of overfitting; however, caution must still be used to avoid misinterpreting that the model has worked better than expected [36].

Person reliability, the IRT equivalent to Cronbach’s alpha, is used to evaluate the internal consistency of the measure. Low values (<0.8) suggest that the measure has too few items or reduced variability in responses (i.e., there are few people with responses in the high or low ranges, indicating more extreme abilities).

To distinguish high performers (in swallowing) from low performers (in swallowing), person separation determines whether the test separates the sample into sufficient levels. When identified as accidental responses, outliers are managed using person separation. For clusters that represent true performances, people are classified using the person separation index (PSI)/strata (4* person separation +1/3). When person separation is low, it can be assumed that the measure is not sensitive enough to separate low and high performers. Reliability values of 0.5, 0.8, and 0.9, respectively, indicate separation into only one or two levels, 2–3 levels, and 3–4 levels [19]. To consistently identify three performance levels, a PSI/strata of 3 is required (the minimum level to attain a reliability of 0.9). An item hierarchy with <3 levels (high, medium, low) is verified using the item reliability. If item reliability is <0.9, then the sample is too small to confirm the measure’s construct validity (item difficulty).

### 2.6. Differential Item Analysis

A differential item functioning (DIF) analysis was performed to examine whether the scale items were used in the same way by all groups. DIF occurs when a characteristic other than the swallowing difficulty being assessed influences the rating of an item [36]. The DIF analysis was performed on all 25 items. We tested DIF in variables where we expected DIF (e.g., OD vs. no OD) and in variables where we did not expect DIF (e.g., sex). The sample was categorised by age (18–39 years vs. 40–59 years vs. 60–69 years vs. 70–79 years vs. >80 years), participant category (OD vs. no OD), sex (male vs. female), diagnostic category (neurological disorders vs. head and neck oncology vs. other disorders), and swallowing difficulty according to FOIS (nothing by mouth vs. tube dependent with minimal attempts of food or liquid vs. tube dependent with consistent oral intake of food or liquid vs. total oral diet of a single consistency vs. total oral diet with multiple consistencies, requiring special preparation or compensations vs. total oral diet with multiple consistencies without special preparation, but with specific food limitation vs. total oral diet with no restrictions).

These were variables of interest based on the current literature about OD. In addition, given that the DHI is a measure of swallowing difficulties, we needed to establish if it could detect differences in performance for those with and without swallowing difficulties, as we would expect this would impact their DHI scores [24]. Patients with neurological disorders (e.g., stroke, acquired brain injury, Parkinson’s disease, multiple sclerosis, cerebral palsy, or Alzheimer’s disease; [37]), head and neck cancer [38], and other disorders (e.g., structural deficits of the oral cavity, pharynx, or larynx; [39]) have been found to have poorer swallowing outcomes.

A significant DIF on a large number of items can indicate item bias. DIF based on age would be expected for older patients [40]. In terms of sex, previous research found that men and women experience similar rates of swallowing difficulty; as such, we do not expect DIF [41]. Swallowing difficulty as classified using the FOIS is expected to show DIF for those with more severe swallowing difficulty [42], as well as DIF for those diagnosed with OD using VFSS or FEES, compared to those without OD [5].

Differential item functioning contrast refers to the difference in difficulty of the item between both groups. Concerning the hypothesis ‘*this item has the same difficulty for two groups*’, DIF is noticeable when the DIF contrast (the reporting of the effect size in Winsteps) is at least 0.5 logits with a *p*-value < 0.05. The combination of DIF contrast (of at least 0.5 logits) and the *p*-value (<0.05) needs to be present, as statistical significance can be affected by sample size, and the sample size may not be large enough to exclude the possibility of being accidental [19]. Inspection results of the direction of the logits in the DIF contrast scores indicate the difficulty of the item in comparison to what was expected (i.e., positive logits indicate that the item was more difficult than expected [lower scores] and negative logits indicate that the item was easier (higher scores)). In determining DIF when comparing more than two groups (i.e., age, diagnoses, FOIS levels, and DHI severity) with the hypothesis ‘*this item has no overall DIF across all groups*’, the chi-square statistic and *p*-value < 0.05 are used [19]. There are two DIF methods used within Winsteps. The Mantel method is used for polytomous data, which are complete or almost complete. The Mantel–Haenszel method is used for uniform DIF analysis of complete or incomplete dichotomous data; for incomplete or sparse data, it uses a logistic uniform DIF method to estimate the difference between the Rasch item difficulties for the two groups, holding everything else constant. To overcome the limitation of incomplete data, Mantel/Mantel–Haenszel in Winsteps are (log-)odds estimators of the DIF size and significance based on the cross-tabulation of the observations of the two groups and use theta to stratify the data. Mantel and Mantel–Haenszel do not require a large sample [43], so they are suitable for our sample size. Winsteps also employs a non-uniform DIF logistic technique and a graphical non-uniform DIF approach. We used the Mantel and Mantel–Haenszel tests as they are considered the most authoritative for DIF analyses of dichotomous and polytomous variables [33].

### 2.7. Dimensionality of the Scale

There are a number of ways to assess dimensionality, including (a) using negative point-biserial correlations to identify problematic items; (b) using Rasch fit indicators to identify misfitting items or persons; and (c) employing Rasch factor analysis using principal component analysis (PCA) on the standardised residuals [44]. A PCA of residuals checks the number of principal components to confirm that there are no second or further dimensions after the intended or Rasch dimension is removed. Where residuals for pairs of items are uncorrelated and normally distributed, it can be assumed that no second dimension is present. To determine if further dimensions in the residuals are present, the following criteria are recommended: (a) the Rasch factor uses a cut-off >60% of the explained variance; (b) on first contrast, an eigenvalue of <3 (equivalent to three items) is used; and (c) a first contrast of <10% of the explained variance is used [19].

Distributions of a person’s abilities and item difficulties are represented using the person–item dimensionality map, using a logit scale framework. For this paper, person ability refers to a person’s self-rated ability to swallow. Items on the DHI that are rated with such infrequency, because very few people with swallowing problems will give these items a high rating, will be classified as “difficult” items. In contrast, “easy” items might refer to aspects of swallowing that occur regularly and will receive high self-ratings. Where two or more items represent similar difficulty, they will be placed in the same location on the logit scale. Gaps in the item difficulty continuum are identified when persons are represented with no corresponding item. Another indication of the overall distribution is the person measure score, using a mean measure score of 50 to determine the location on the person item map. A centralised item mean score of lower than 50 implies that people in the sample were more able than the level of difficulties in the items; higher than 50 indicates a lower ability than the mean item difficulty.

## 3. Results

The sample of 256 records from people at risk of OD was used for Rasch analyses, thus meeting the COSMIN criteria of an adequate sample size (more than five times the number of items [5 × 25 = 125], and a minimum sample of 100) [32]; 53.1% were male, and 46.9% were female, with an overall mean age of 65.2 years (SD 14.2; range 18–96 years). A total of 188 patients with confirmed OD and 68 patients without OD were included. About one-third of patients were diagnosed with neurological disorders, one-third with head and neck oncology, and the remaining patients reported dysphagia due to other medical causes (e.g., dysphagia after surgery or presbyphagia). Oral intake data (FOIS) and dysphagia severity (DHI) data showed a wide spread of swallowing ability. No data were missing except for the DHI severity scale (missing data: 13/256; 5.1%). The participants’ demographic information is reported in Table 2.

### 3.1. Rating Scale Validity

The Dysphagia Handicap Index (DHI) is a 25-item measure of three domains of quality of life (QoL) related to the physical aspects of dysphagia (9 items), functional aspects (9 items), and emotional aspects (7 items). The respondents rate the extent to which each statement applies to them with scores of (0) for never, (1) for sometimes, and (2) for always. This results in a maximum score of 50, with higher scores indicating poorer swallowing ability. Respondents also rate their perception of the severity of their swallowing difficulty on a scale from 1 (normal) to 7 (severe problems). We first examined the instrument overall, followed by individual analyses of the three subscales, and finally, we completed analyses to test the removal of items to determine if this improved the fit to the model.

We first examined the response category, item and person fit, dimensionality, and DIF for the DHI, and then for each of the subscales, physical, functional, and emotional aspects, and then finally examined the effect of removing each of the most misfitting items in the overall scale.

#### Category Order

**Figure 2:**
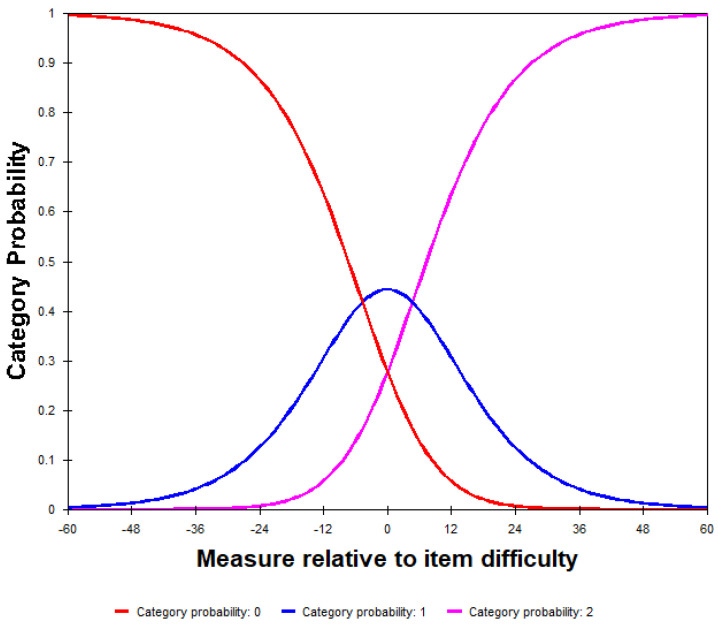
Rating scale validity.

The examination of the response category for the overall instrument revealed that as the category order increased (from 0 to 2), all fit statistics were in the acceptable range (Z-STD = 0.7–1.4), with the average measure scores increasing monotonically, indicating three distinct, ordered categories (see Table 3 and Figure 2). The Andrich thresholds reflect the relative frequency of use of the categories, and these were not disordered, but the step difficulty in the categories advanced by >5 logits between categories 1 and 2 (+4.69) (4.69 − (−4.69) = 9.38 logits), indicating a potential gap in the measure of the variable (i.e., in the rating category labels).

We then examined the category order for each of the three subscales. Average measures for the physical and functional subscales increased monotonically, and the examination of the Andrich thresholds revealed they were not disordered but increased by <5 logits between categories 0 and 1 on the functional subscale (−4.80), but by >5 logits on the physical subscale (−7.54). The step difficulty increased by >5 logits between categories 1 and 2 on the functional subscales (+4.80) (4.80 − (−4.80) = 9.60 logits) and on the physical subscale (+7.54) (7.54 − (−7.54) = 15.08 logits). For the emotional subscale, the average measure did not increase monotonically, and the Andrich thresholds were ordered but increased by >5 logits between 0 and 1 (−8.23) and between 1 and 2 (+8.23) (8.23 − (−8.23) = 16.46 logits). The examination of the category fit statistics revealed no categories in the misfit range.

### 3.2. Person and Item Fit

The summary item and person ability infit and outfit statistics for the 25-item scale were examined (see Table 4). There was a good item reliability estimate (0.97) of items with a separation of 5.76, and person reliability was 0.91. The person separation index (PSI) was 3.23, indicating that persons were reliably separated into at least three distinct groups based on the strata of abilities. When examining the subscales’ item reliability estimates, they were also good (0.96–0.98), with item separation ranging from 4.89 to 7.72, but person reliability was moderate (0.72–0.82). The PSIs were poor, ranging from 1.6 (emotional) and 1.76 (physical), indicating that persons were not separated into at least two levels. For the functional subscale, it was 2.13. An examination of the point measure correlations for all 25 items revealed that they were all positive, indicating that all items contributed to the measurement of the latent variable. This was also the case for point measure correlations of the subscales.

Item fit statistics are provided in Table 5. The MnSq infit and outfit statistics should be close to 1 with an acceptable range of (0.70–1.4), and they are reported as overfitting if <0.7 and underfitting if >1.4 (Bond and Fox 2015 [36]). To fit the model, infit and outfit reported as Z-STD statistics (standardised fit statistics) have an expected outcome of 0 with an acceptable range of ±2. Values exceeding +2 are reported as *underfit* and as *overfit* if they exceed −2 (Bond and Fox, 2015). All *infit* MnSqs were in the acceptable range except for *underfit* for item 22 (F), and it also had *underfit* Z-STD statistics. *Infit* Z-STD values were also *underfitting* for items 3 (P), 5 (P), 7 (F), and 24 (P). *Infit* Z-STD values were *overfitting* for items 2 (P), 12 (E), 13 (E), 14 (F), 15 (F), 16 (F), and 23(F). *Outfit* MnSqs were in the desired range except when *underfitting* in items 3 (P) and 24 (P), and *overfitting* in items 12 (E), 14 (F), 16 (F), and 18 (E). *Outfit* Z-STD values were also *underfitting* (>2) for items 1 (P), 3 (P), 7 (F), 17 (E), 20 (P), 21 (E), and 24 (P) and *overfitting* for items 12 (E), 13 (E), 14 (F), 15 (F), 16 (F), 18 (E), and 23 (F). When mean squares are acceptable, underfitting and overfitting Z-STD values can be ignored [19].

When examining the person fit statistics, 49 persons had some underfitting MnSqs or Z-STD scores. Twenty-nine persons had both underfitting infit and outfit MnSqs (>1.4). Overall, infit MnSq scores for 35 persons and 22 infit Z-STD scores were underfitting. Sixteen persons had both underfitting infit and outfit Z-STD scores. Overall, infit Z-STD scores were underfitting for 22 persons and the Z-STD outfit scores for 21 persons were underfitting. Infit statistics explain performance better because outfit statistics are sensitive to outlying scores. Too much variation in the responses results is underfitting (MnSq >1.4; Z-STD > 2), and this is the biggest threat to the measure because it can degrade the model (Bond and Fox 2015 [36]). We then examined the item and person fit statistics for each subscale.

#### 3.2.1. Physical Subscale

Items 1, 2, 3, 4, 5, 11, 20, 24, and 25 comprised the physical subscale. All items had MnSq infit and outfit scores in the desired range except item 5 (infit, 1.46; outfit, 1.48), which was also underfitting for both the MnSq (5.05) and Z-STD (4.26) outfit scores. An overfitting Z-STD score (−2.23) was also evident on item 1 and on items 2 (−3.43) and 4 (−2.93), which also had underfitting outfit Z-STD scores (−2.45 and −2.45, respectively).

Fifty-one persons had at least one misfitting infit or outfit statistic. In total, 38 people had underfitting infit and outfit MnSq scores, 12 persons had underfitting Z-STD infit and outfit scores, and 12 persons had both infit and outfit MnSq and Z-STD scores that were underfitting.

#### 3.2.2. Functional Subscale

Items 6, 7, 9, 10, 14, 25,16, 22, and 23 comprised the functional Subscale. All items had MnSq infit scores in the desired range except item 22 (1.41), and the Z-STD infit score was also underfitting (3.18). Items 7 and 9 had MnSq outfit scores (1.62 and 1.59, respectively) and overfitting Z-STD outfit scores (4.26 and 2.86, respectively). Item 6 had both overfitting infit (−2.02) and outfit (−2.05) Z-STD scores, as did item 23 with an infit Z-STD score (−5.03 and outfit Z-STD of −4.62), which was also underfitting on both the MnSq (5.05) and Z-STD (4.26) outfit scores. Overfitting Z-STD scores were also evident on item 1 (−2.23), item 2 (−3.43), and item 4 (−2.93), which also had underfitting outfit Z-STD scores (−2.45 and −2.45, respectively). Items 15 and 16 had overfitting infit Z-STD scores (−2.64 and −2.42, respectively).

Forty-three persons had at least one misfitting infit or outfit statistic. In total, 21 people had underfitting infit and outfit MnSq scores, 10 persons had underfitting Z-STD infit and outfit scores, and 10 persons had both infit and outfit MnSq and Z-STD scores that were underfitting.

#### 3.2.3. Emotional Subscale

Items 8, 12, 13, 17, 28, 29, and 21 comprised the emotional subscale. All items had MnSq infit scores in the desired range, but item 21 had an underfitting outfit MnSq (1.44), and both the infit Z-STD score (3.06) and outfit (3.38) were underfitting. Item 8 had an overfitting Z-STD infit score (2.38), and both the infit and outfit Z-STD scores for item 12 (−3.96 and −3.46, respectively) and item 18 (−2.87 and −2.72, respectively) were overfitting.

Sixty-three persons had at least one misfitting infit or outfit statistic. Thirty-five persons had underfitting infit and outfit MnSq scores, five persons had both underfitting Z-STD infit and outfit scores, and five persons had both infit and outfit MnSq and Z-STD scores that were underfitting.

### 3.3. Differential Item Functioning

Differential Item Functioning (DIF) analysis enabled the examination of potential contrasting item-by-item profiles associated with the following: sex, age, diagnostic category, OD or no OD, FOIS score, and DHI severity score. The summary of the DIF analysis for all 25 items as the overall scale is presented in Table 6. Significantly different responses were most frequently observed for six P items, five F items, and one E item. Significant DIF for sex was observed on two items: 3 (P) and 24 (P); for age on item 4 (P); for diagnostic category on items 24 (P), 6 (E), 20 (P), 22 (F), and 23 (F); for OD vs. no OD on item 24 (P); for FOIS on items 1 (P), 6 (E), 15 (F), 16 (F), 17 (F), 20 (P), 22 (F), and 23 (F); and DHI severity on items 3 (P), 20 (P), and 25 (P).

Differential item functioning was then examined for each of the subscales. As with the overall scale, no items showed significant DIF for all variables; however, three P items (3, 24, and 25) showed DIF on sex, and three P items (1, 2, and 20) showed significant DIF on diagnostic category. On the physical subscale, significant DIF was also evident for age on item 4, for OD vs. no OD on item 24, for FOIS on item 11, and for DHI severity on item 11. For the functional subscale, DIF was evident only for age and DHI severity on item 7, for DHI severity only on item 10, and for FOIS score on items 9, 22, and 23. No DIF was evident on the emotional subscale except for OD vs. no OD and FOIS on item 17.

### 3.4. Dimensionality

The dimensionality of the overall scale of 25 items was examined using the principal component analysis (PCA) of the residuals (Table 7 and Table 8). Contrasts in the item residuals are examined for dimensions that are not explained by the Rasch dimension. The Rasch dimension explained 42.9% of the variance, with >40% indicating a strong measurement of dimension [19]. The examination of the explained variance showed that the item measures (22.3%) explained slightly more of the variance than the person measures (20.7%). However, the unexplained variance (57.1%) was greater than the explained variance. The raw variance explained by the items was only about three times the variance explained by the first contrast (7.8%), indicating a noticeable second dimension. The first contrast had an eigenvalue of 3.43, which is greater than the value (two eigenvalue units) confirming that there is a second dimension and the eigenvalue of the second contrast (2.11), explaining 4.8% of the variance, which is the smallest amount that could indicate the possibility of a third dimension. The PCA divided the items into two groups: one with the Rasch dimension items 1, 2, 3, 4, 11, 20, 24, and 25 from the physical (P) subscale and 17,19, 21 from the emotional (E) subscale and another with a second dimension with items 6, 7, 9, 10, 14, 15, 16, 22, and 23 from the functional (F) subscale and items 8, 12, 13, and 18 from the Emotional (E) subscale. This would suggest, based on the theoretical logic for QoL, that for people with dysphagia, QoL is affected by physical symptoms and the functional impact on daily life. However, the results related to the dimensionality of the DHI suggest that the emotional impact is intertwined with both physical symptoms (e.g., having an emotional response [fear] to choking) and also in response to the functional impact (e.g., having an emotional response [depression] to appearing in public). As indicated earlier, the point measure correlations were all in a positive direction, indicating that all items contributed to the measurement of the latent variable and should, therefore, be retained.

**Figure 3:**
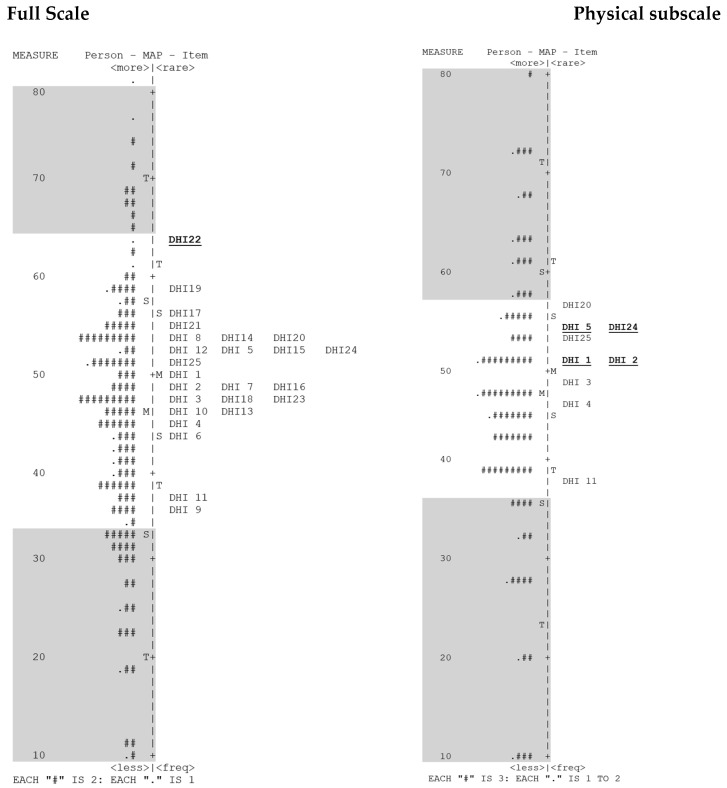

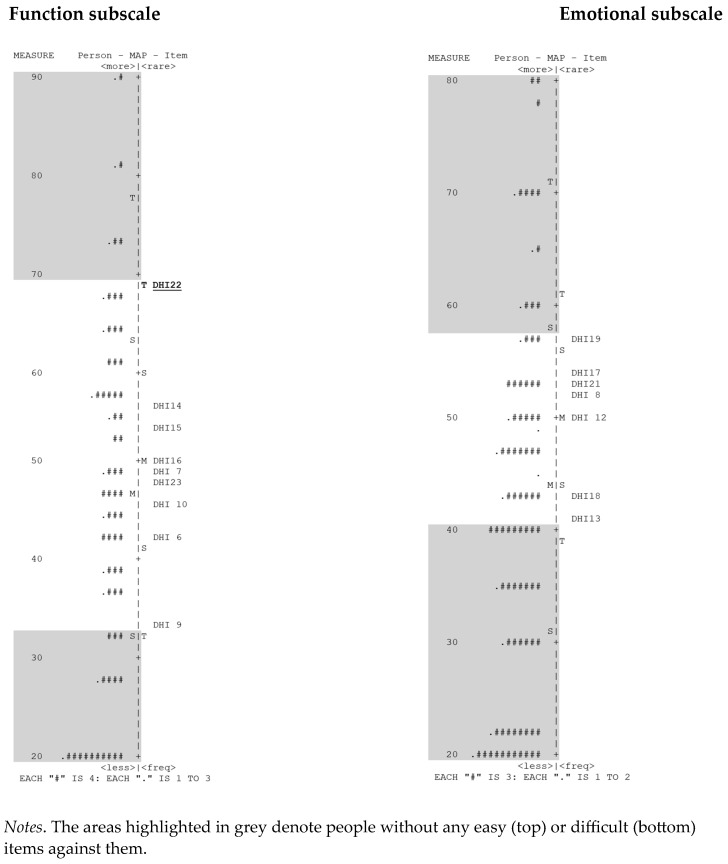
Person–Item map.

As presented in Figure 3, the person–item map showed that (a) there was a need for more easy and more difficult items, (b) that many people were not aligned against items, and (c) that there was very little item redundancy evident from items aligning at the same level. Items 8 (E), 14 (F), and 20 (P) aligned; 5 (P), 12 (E), 15 (F), and 24 (P) aligned; 2 (P), 7 (F), and 16 (F) aligned; 3 (P), 18 (E), and 23 (F) aligned; and items 10 (F) and 13 (E) aligned. However, of these, items 5 and 24 were both P items, so one was potentially redundant, and 7 and 16 were both F items, so one was potentially redundant. The other alignments can be explained because they are items belonging to differing subscales.

We also examined the dimensionality of each of the subscales separately. On the physical subscale, the Rasch dimension explained 38.4% of the variance, with persons and items explaining 18.5% and 20.3%, respectively. There was no evidence of a second dimension, with the first contrast variance being less than two eigenvalue units. On the functional subscale, 54.2% of the variance was explained by the Rasch dimension, with person and item variances of 28.2% and 26%, respectively, and no evidence of a second dimension. On the emotional subscale, 44.7% of the variance was explained by the Rasch dimension, with person and item variances of 23.8% and 20.9%, respectively, and no evidence of a second dimension. As with the overall measure, there was a need for more easy and more difficult items in each subscale. Many people were not aligned against items, and the only potential redundancies were in the physical subscale, with items 5 and 24 aligning at the same level (as observed in the overall scale) and items 1 and 2 aligning at the same level.

This process was then repeated with the removal of the most misfitting items—18 (I feel handicapped because of my swallowing (E)), 24 (I feel a strangling sensation when I swallow (P)), and 3 (my mouth is dry (P))—separately and then of combinations of items 3 and 24 and items 3, 18, and 24. Even though item 22 (I must eat another way (e.g., feeding tube) because of my swallowing problem (F)) was misfitting, it was the only difficult item and was therefore not removed in these analyses. No significant changes were evident, and all models still indicated a second dimension (Table 7 and Table 8) or measures of the two components of QoL impact, physical and functional, with emotional as a third component related to both physical and functional items. However, examining the person-item map revealed that the removal of both items 3 and 24 resulted in only items 7 and 16 showing redundancy.

## 4. Discussion

### 4.1. Summary Statistics

The summary statistics for item and person ability for all 25 items were good (i.e., high item and person reliability). When examining the subscales, item reliability estimates were good, but person reliability was moderate with poor person separation indices. As a result, people may not be separated into different levels (i.e., high versus low performers in relation to swallowing), supporting the need for more easy and more difficult items.

### 4.2. Rating Scale

When examining how the rating scale was used for the overall DHI, there was no disordering in the categories, and all fit statistics were within an acceptable range. However, step difficulty between the categories indicated a potential gap in the measure of the variable. An examination of the category fit statistics per subscale revealed no categories in the misfit range except for the emotional subscale, and gaps related to step difficulty in all three subscales were confirmed. Increasing the number of categories (i.e., response options) and providing clear descriptions of how categories differ from each other can help resolve these findings.

### 4.3. Person and Item Fit

Overall, more misfitting was evident in the Z-STD statistics, with only one underfitting MnSq infit statistic. Outfit MnSqs outside the acceptable range were mainly overfitting and not in contradiction to the outfit Z-STD scores, with overfitting being less of a threat to the model. However, care needs to be taken so that this is not misinterpreted as ‘*the model is working better than expected*’.

When using the scale as a whole, all items except item 22 had acceptable mean square infit statistics, and therefore, the underfitting and overfitting of the Z-STD scores are considered less important. Outfit MnSqs were overfitting for four items, but the outfit Z-STD scores were also overfitting. The overfitting of MnSq and Z-STD is less concerning than underfitting. Outfit statistics are unweighted, so they are often regarded as less important than infit statistics as they are more sensitive to outliers. Although they show that the data were more predictable than the model, they do not usually degrade the model. Further, although some items had an MnSq infit or outfit score outside the desired range, suggesting their removal, additional analyses assessing the measure’s dimensionality recommend that the measure may be improved as a two-dimensional model, with these questions retained but with different wording. Item 22 had infit MnSq and Z-STD scores that were underfitting, but this is likely due to it being about needing to use an alternative means of feeding (i.e., feeding tube). So, it should be retained as it would likely perform better with a larger sample that included more people using feeding tubes.

### 4.4. Differential Item Functioning

Differential item functioning analysis was used to examine the potential contrasting item-by-item profiles associated with sex, age, the presence or absence of a confirmed diagnosis of OD, medical diagnosis, FOIS, and DHI severity. Overall, the DIF results indicate good performance at an item level. Theoretically, DIF would be expected on most variables, but not for sex. The most obvious DIF was found for FOIS, suggesting a more optimal representation for the functional manifestation of swallowing problems. In contrast, the presence of dysphagia presented only with marginal DIF. The DIF domains age, medical diagnosis, and DHI severity also showed minor DIF, of which the limited DIF for age could be due to a limited distribution across age groups (i.e., 71.1% of participants were ≥60 years).

### 4.5. Dimensionality

The PCA of residuals performed to examine the dimensionality of the overall DHI measure indicated that the DHI consisted of two dimensions in contrast to the three dimensions as suggested by Silbergleit, Schultz [24]; each dimension consisted of items either from the functional or physical subscale, supplemented with items originating from the emotional subscale. The person–item dimensionality map indicated little item redundancy, but an obvious need to generate more easy and difficult items.

Because Rasch analyses could not confirm the three-dimensional nature of the DHI, it is recommended to avoid the use of subscales and consider only the full measure until dimensionality is addressed through future instrument redevelopment. During the redevelopment process, items from the emotional subscale may be distributed across the other two domains and/or reworded to reflect that physical and functional domains have an emotional element, rather than emotion being a separate dimension.

### 4.6. Future Recommendations

Failing to reproduce the three subscales suggests that the DHI does not meet content validity criteria. Future studies should focus on meeting criteria for all three aspects of content validity: (1) relevance (i.e., the degree to which all items of a measure are relevant for the construct of interest within a target population and purpose of use); (2) comprehensiveness (i.e., the degree to which all key concepts of the construct are included in a measure); and (3) comprehensibility (i.e., the degree to which items of a measure are easy to understand for respondents) [45].

The redevelopment of the DHI should also address the rephrasing and regrouping of misfitting items and the need for the generation of new, easy, and difficult items to improve the separation of people into different levels of low versus high performance (i.e., a low versus high degree of disability on patient’s quality of life). In supporting a two-dimensional model, all emotion items should be reworded to rather reflect an emotional response to a physical or a functional challenge. For example, item 12, ‘*I feel depressed because I can’t eat what I want*’ can be changed to ‘*Not being able to eat what I want makes me feel depressed*’, which then becomes a functional item with an emotional component. After the redevelopment of the DHI, the revised measure’s psychometric properties, including its dimensionality, must be determined again using CTT and IRT analyses in preferably the same, larger sample sizes, meeting current international standards for instrument development.

As solving the current psychometric issues surrounding the DHI is challenging, the development of a new measure that ensures good content validity may be preferred. During the instrument development of patient self-report measures for dysphagia, careful consideration should be given to which constructs should be targeted and whether including the constructs *functional health status* and *health-related quality of life* may suffice [7]. In this context, functional health status refers to the impact of dysphagia on the ability to perform tasks in multiple domains (including physical, social, role, and psychological functioning) and aims to quantify the symptomatic severity and (loss of) function due to dysphagia and/or treatment and the impacts on daily life as experienced by patients at a particular point in time [7]. HR-QoL refers to the unique personal perception of someone’s health, taking into account social, functional, and psychological issues [9].

A final consideration is the naming of the measure: the Dysphagia Handicap Index. When redeveloping the DHI, one may consider changing the measure’s name, as the term ‘handicap’ is perceived as outdated and offensive [46]. Instead, preference should be given to recommended language as suggested by, for example, the United Nations [46], which refers to ‘persons with disabilities’ and targeting patients’ *capabilities* over *disabilities*.

## 5. Conclusions

In general, previous studies using CTT to determine the psychometric properties of the DHI confirmed its validity and reliability [24]. However, our current findings using IRT seem to contradict the results of these CTT studies to some degree and highlight the need for continuous instrument development. The main weakness of the DHI is related to the failure to reproduce its three subscales, suggesting that the DHI does not meet the content validity criteria. The DHI has two dimensions, not three, and the items from the emotional subscale should be reworded and integrated with the functional and physical subscales. The redevelopment of the DHI should focus on meeting all criteria for good content validity, address the rephrasing and regrouping of misfitting items, and include new, easy, and difficult items to improve the separation of people into different levels of swallowing ability. Given the complexity of addressing these issues, the development of a new measure that ensures good content validity may be preferable.

## Figures and Tables

**Table 2:** Participant demographics.

Participant Characteristics	Oropharyngeal Dysphagia Group (*n* = 188)	No Oropharyngeal Dysphagia Group (*n* = 68)	Combined Groups (N = 256)
**Sex**: *n* (%)			
Male	103 (54.8%)	33 (48.5%)	136 (53.1%)
Female	85 (45.2%)	35 (51.5%)	120 (46.9%)
**Age** (≥18 years):			
MN (SD)	66.4 (13.8)	61.8 (14.8)	65.2 (14.2)
Range	18–96	18–88	18–96
**Age group:** *n* (%)			
18–39	7 (3.7%)	6 (8.8%)	13 (5.1%)
40–59	42 (22.3%)	19 (27.9%)	61 (23.8%)
60–69	52 (27.7%)	18 (26.5%)	70 (27.3%)
70–79	58 (30.9%)	22 (32.4%)	80 (31.3%)
≥80	29 (15.4%)	3 (4.4%)	32 (12.5%)
**Medical diagnosis:** *n* (%)			
Neurological disorders	60 (31.9%)	16 (23.5%)	76 (29.7%)
Head and Neck oncology	59 (31.4%)	9 (13.2%)	68 (26.6%)
Other	69 (36.7%)	43 63.2%)	112 (43.8%)
**FOIS**			-
All levels: Med (25; 75%)	6 (5; 6)	7 (7; 7)	
*Per level: n (%)*			
1. Nothing by mouth	14 (7.4%)	-	-
2. Tube dependent with minimal attempts of food or liquid	12 (6.4%)	-	-
3. Tube dependent with consistent oral intake of food or liquid	6 (3.2%)	-	-
4. Total oral diet of a single consistency	11 (5.9%)	-	-
5. Total oral diet with multiple consistencies, requiring special preparation or compensations	44 (23.4%)	-	-
6. Total oral diet with multiple consistencies without special preparation, but with specific food limitation	58 (30.9%)	-	-
7. Total oral diet with no restrictions	111 (22.9%)	-	-
**DHI severity** (missing data = 13)	(*n* = 181)	(*n* = 62)	(N = 243)
1. No difficulty at all	1 (0.6%)	8 (12.9%)	9 (3.7%)
2.	8 (4.4%)	8 (12.9%)	16 (6.6%)
3.	14 (7.7%)	2 (3.2%)	16 (6.6%)
4. Somewhat of a problem	36 (19.9%)	14 (22.6%)	50 (20.6%)
5.	38 (21.0%)	10 (16.1%)	48(19.8%)
6.	50 (27.6%)	15 (24.2%)	65 (26.7%)
7. The worse problem you could have	34 (18.8%)	5 (8.1%)	39 (16.0%)

*Notes*. FOIS = Functional Oral Intake Scale; DHI = Dysphagia Handicap Index; MN = Mean; Med = Median; SD = Standard Deviation.

**Table 3:** Category function.

Category	N	%	Average Measures	Infit MnSq	Outfit MnSq	Andrich Thresholds
0	2759	43	−13.52	1.05	1.10	None
1	1983	31	−2.83	0.93	1.00	−4.69
2	1656	26	8.49	0.97	1.01	4.69

*Note*: Missing data = 2; 0.03%.

**Table 4:** Item and person summary statistics.

Analysis	Scales	Item/ Person	Rel	Sep	PSI *	Mean Measure	Model *SE*	MnSq	Z-STD	MnSq	Z-STD
**1**	**All 25 items**	**Person**	0.91	3.23	4.64	45.60	3.56	1.01	−0.08	1.04	−0.03
		**Item**	0.97	5.76	-	50.00	1.03	1.01	−0.15	1.04	0.12
**2**	**Physical**	**Person**	0.76	1.76	2.68	47.51	5.73	1.01	−0.05	1.02	−0.05
	**Scale**	**Item**	0.96	4.89	-	50.00	1.05	1.00	−0.21	1.02	0.07
**3**	**Function**	**Person**	0.82	2.13	3.17	47.40	6.27	1.00	−0.04	1.03	0.02
	**Scale**	**Item**	0.98	7.72	-	50.00	1.16	1.02	−0.16	1.03	−0.25
**4**	**Emotional**	**Person**	0.72	1.61	2.48	44.16	6.95	1.01	−0.04	1.04	0.00
	**Scale**	**Item**	0.96	4.61	-	50.00	1.19	0.99	−0.28	1.04	0.11

***Notes*.** * PSI, Person Separation Index/Strata; PSI = [4 × Person Separation + 1]/3. A person strata of, “3” (the minimum level to attain a reliability of 0.90) implies that three different levels of performance can be consistently identified using the test for samples like that tested; Rel = reliability; Sep = separation; bold text and thicker lines reflect that each analysis generates both person and item statistics.

**Table 5:** Individual item fit statistics and principal component analysis for subscales.

	All 25 Items					Physical Subscale					Function Subscale					Emotional Subscale				
	Infit		Outfit			Infit		Outfit			Infit		Outfit			Infit		Outfit		
Items	MnSq	Z-STD	MnSq	Z-STD	PTM Corr.	MnSq	Z-STD	MnSq	Z-STD	PTM Corr.	MnSq	Z-STD	MnSq	Z-STD	PTM Corr.	MnSq	Z-STD	MnSq	Z-STD	PTM Corr.
1	0.96	−0.55	1.24	**2.06**	0.46	0.83	−2.23	0.97	−0.28	0.56	-	-	-	-	-	-	-	-	-	-
2	0.80	**−2.68**	0.86	−1.35	0.59	0.75	−3.43	0.79	−2.45	0.64	-	-	-	-	-	-	-	-	-	-
3	1.28	**3.38**	**1.53**	**4.41**	0.46	1.08	1.01	1.15	1.65	0.58	-	-	-	-	-	-	-	-	-	-
4	1.12	1.46	1.05	0.54	0.59	0.99	−0.08	0.94	−0.65	0.66	-	-	-	-	-	-	-	-	-	-
5	1.25	**2.94**	1.17	1.42	0.55	**1.46**	**5.05**	**1.48**	**4.26**	0.51	-	-	-	-	-	-	-	-	-	-
6	0.99	−0.05	0.94	−0.53	0.68	-	-	-	-	-	0.82	**−2.02**	0.75	**2.05**	0.78	-	-	-	-	-
7	1.24	**2.87**	1.37	**3.18**	0.52	-	-	-	-	-	1.38	**3.79**	**1.62**	**4.26**	0.62	-	-	-	-	-
8	1.16	1.87	1.18	1.35	0.55	-	-	-	-	-	-	-	-	-	-	1.23	**2.38**	1.20	1.78	0.66
9	1.02	0.29	1.01	0.16	0.64	-	-	-	-	-	1.31	**2.79**	**1.59**	**2.86**	0.66	-	-	-	-	-
10	1.01	0.18	0.99	−0.08	0.64	-	-	-	-	-	1.11	1.19	1.10	0.79	0.71	-	-	-	-	-
11	1.09	1.09	1.03	0.28	0.63	1.10	1.17	0.96	−0.35	0.66	-	-	-	-	-	-	-	-	-	-
12	0.70	**−4.18**	**0.66**	**−3.17**	0.69	-	-	-	-	-	-	-	-	-	-	**0.69**	**−3.96**	**0.68**	**−3.46**	0.78
13	0.75	**−3.48**	0.70	**−3.21**	0.72	-	-	-	-	-	-	-	-	-	-	1.03	0.39	1.16	1.51	0.72
14	0.80	**−2.65**	**0.69**	**−2.75**	0.67	-	-	-	-	-	0.97	−0.26	0.90	−0.67	0.69	-	-	-	-	-
15	0.84	**−2.05**	0.76	**−2.17**	0.67	-	-	-	-	-	0.78	**−2.64**	0.75	−1.93	0.74	-	-	-	-	-
16	0.76	**−3.31**	**0.69**	**−3.23**	0.71	-	-	-	-	-	0.80	**−2.42**	0.77	−1.91	0.76	-	-	-	-	-
17	0.90	−1.16	1.33	**2.24**	0.55	-	-	-	-	-	-	-	-	-	-	0.86	−1.56	1.01	0.16	0.70
18	0.70	**−4.35**	**0.68**	**−3.43**	0.72	-	-	-	-	-	-	-	-	-	-	0.77	**−2.87**	0.75	**−2.72**	0.78
19	1.06	0.68	1.04	0.28	0.52	-	-	-	-	-	-	-	-	-	-	1.05	0.56	1.01	0.13	0.65
20	1.06	0.77	1.30	**2.20**	0.47	0.87	−1.65	0.92	−0.76	0.60	-	-	-	-	-	-	-	-	-	-
21	1.15	1.69	1.35	**2.40**	0.46	-	-	-	-	-	-	-	-	-	-	1.30	**3.06**	**1.44**	**3.38**	0.59
22	**1.48**	**3.90**	1.18	0.93	0.46	-	-	-	-	-	**1.41**	**3.18**	1.26	1.03	0.56	-	-	-	-	-
23	0.81	**−2.65**	0.74	**−2.72**	0.71	-	-	-	-	-	**0.61**	**−5.03**	**0** **.53**	**−4.62**	0.81	-	-	-	-	-
24	1.25	**2.88**	**1.54**	**3.96**	0.44	1.09	1.15	1.15	1.51	0.55	-	-	-	-	-	-	-	-	-	-
25	0.94	−0.69	1.01	0.10	0.56	0.78	**−2.93**	0.79	**−2.35**	0.65	-	-	-	-	-	-	-	-	-	-

*Notes.* MnSq values outside the acceptable range of 0.7–1.4 and outfit Z-STD values that exceed ±2 are interpreted as not fitting the Rasch model [36]; PTM Corr. = point measure correlations; values that are in bold are outside the acceptable range and do not fit the Rasch model.

**Table 6:** Summary of DIF analysis.

	Sex			Age			OD vs. No OD		
Items	Mantel–Haenszel Prob.	Prob.	DIF Contrast (Effect Size) ^&^	Summary DIF Chi-Squared	Prob.	DIF Contrast (Effect Size) ^∧^	Mantel–Haenszel Prob.	Prob.	DIF Contrast (Effect Size) ^#^
1	0.0219	0.8824	2.14	1.7218	0.7866	−4.57	0.3165	0.5737	−4.98
2	0.4132	0.5204	1.98	0.4891	0.9746	−1.15	0.2510	0.6164	−1.85
3	4.8669	0.0274 *	6.85 *	5.6128	0.2295	−3.57	0.0002	0.9897	−4.35
4	0.2014	0.6536	0.22	12.4567	0.0142 *	9.19 *	0.2211	0.6382	−2.84
5	0.0312	0.8597	0.00	4.5205	0.3395	14.08	0.3449	0.5570	−1.54
6	0.6014	0.4380	0.49	6.8033	0.1462	11.80	1.2673	0.2603	6.47
7	1.7715	0.1832	−3.82	6.7215	0.1509	−4.57	1.4411	0.2300	1.14
8	0.0009	0.9761	−0.55	2.5851	0.6291	−4.77	0.2077	0.6486	0.61
9	1.1216	0.2896	−1.85	5.1270	0.2740	0.46	0.3166	0.5737	−0.88
10	0.0597	0.8069	−0.48	8.0170	0.0906	13.02	0.4347	0.5097	1.44
11	0.1059	0.7449	−1.14	6.2638	0.1798	7.12	0.0129	0.9095	2.56
12	0.8468	0.3575	−1.37	2.3896	0.6641	0.92	1.8811	0.1702	0.00
13	0.0373	0.8469	0.66	1.2558	0.8688	1.25	0.8347	0.3609	2.89
14	0.6785	0.4101	−0.92	3.4626	0.4830	−4.26	0.0536	0.8170	4.54
15	0.4824	0.4874	−1.91	1.4846	0.8292	4.67	0.8328	0.3615	0.34
16	0.1101	0.7400	−0.44	8.0518	0.0894	13.90	0.0697	0.7918	4.06
17	0.0623	0.8029	−0.56	5.1775	0.2690	−11.16	1.8024	0.1794	−5.50
18	2.5779	0.1084	−1.36	2.9914	0.5588	−5.21	0.0091	0.9239	3.73
19	1.4001	0.2367	−4.45	3.2293	0.5197	−4.07	2.0638	0.1508	−4.15
20	0.7719	0.3796	1.41	4.7219	0.3165	−8.17	0.0333	0.8553	−5.26
21	0.9496	0.3298	0.22	6.6618	0.1544	−8.41	2.3633	0.1242	1.72
22	0.5081	0.4760	−3.12	9.0681	0.0592	7.28	3.0012	0.0832	17.38
23	0.0064	0.9363	−0.69	5.4813	0.2408	12.56	2.9663	0.0850	9.85
24	5.7798	0.0162 *	7.22 *	6.6305	0.1563	−13.21	6.5539	0.0105 *	−10.13 *
25	0.9615	0.3268	−1.43	7.2009	0.1253	−10.65	0.0156	0.9005	−2.91
	**Diagnostic Category**			**FOIS**			**DHI Severity**		
**Items**	**Summary DIF Chi-Squared**	**Prob.**	**DIF** **Contrast (Effect Size) ^+^**	**Summary DIF Chi-Squared**	**Prob.**	**DIF Contrast (Effect Size)** **^$^**	**Summary DIF Chi-Squared**	**Prob.**	**DIF Contrast (Effect Size) ^£^**
1	5.7028	0.0566	−5.40	19.5947	0.0033 *	−8.00	15.6503	0.0285 *	−12.13
2	3.3906	0.1807	−3.05	4.1668	0.6540	−0.71	6.7506	0.4552	−10.05
3	2.2554	0.3202	−0.08	20.1350	0.0026 *	−7.72	25.6152	0.0006 *	−10.82
4	2.0901	0.3480	3.79	8.9374	0.1769	1.30	11.2011	0.1300	−8.03
5	1.2538	0.5312	−0.09	4.7373	0.5778	4.88	7.9177	0.3398	−0.53
6	6.3553	0.0408 *	4.30	27.9819	0.0001 *	9.57	6.8707	0.4423	−4.19
7	1.8709	0.3888	−3.36	4.7594	0.5749	−4.09	9.4548	0.2215	−23.96
8	0.0916	0.9571	−0.80	7.1079	0.3108	2.67	4.8344	0.6801	−0.53
9	3.6838	0.1559	−2.94	12.6267	0.0493 *	3.36	4.0174	0.7777	15.13
10	0.2233	0.8959	0.97	8.4497	0.2068	9.09	9.1300	0.2433	15.67
11	1.2911	0.5213	−3.21	10.2473	0.1145	5.36	5.4449	0.6057	7.12
12	3.7545	0.1505	2.85	6.8650	0.3333	−0.51	6.8843	0.4409	3.19
13	1.8676	0.3894	2.09	3.7856	0.7056	11.78	6.8097	0.4488	15.67
14	0.9241	0.6278	0.96	3.1711	0.7870	−4.42	12.2744	0.0918	13.78
15	1.9981	0.3646	−1.02	15.3185	0.0179 *	−2.44	0.3943	0.2990	−4.65
16	1.2585	0.5299	2.79	14.8529	0.0214 *	−1.72	9.4284	0.2232	11.82
17	1.9294	0.3775	−2.10	12.6429	0.0490 *	−9.87	3.4994	0.8353	−2.16
18	0.9943	0.6059	2.28	6.6258	0.3566	1.29	11.6305	0.1133	15.68
19	0.0177	0.9920	0.26	9.5248	0.1460	−3.01	3.6010	0.8244	−11.71
20	16.8816	0.0002 *	−0.36	22.1033	0.0012 *	4.40	20.1818	0.0052 *	15.68
21	0.5044	0.7769	−1.05	8.6827	0.1920	−7.73	8.2005	0.3151	−16.65
22	10.7063	0.0046 *	−1.43	56.8543	0.0000 *	0.88	11.7623	0.1086	−14.55
23	7.7838	0.0199 *	6.45	31.6820	0.0000 *	8.13	9.9422	0.1917	9.80
24	30.8835	0.0000 *	−0.76	25.5090	0.0003 *	−0.96	8.2663	0.3096	−0.53
25	1.6022	0.4453	−1.11	10.9412	0.0901	−2.30	14.8209	0.0383 *	3.19

*Notes*. ^&^ Sex (male reference group); **^∧^** 18–39 vs. 40–59 vs. 60–69 vs. 70–79 vs. ≥80 (18–39 reference group); ^#^ OD status (OD reference group); ^+^ neurological disorders vs. head and neck oncology vs. other disorders (neurological disorders reference group); ^$^ FOIS (nothing by mouth reference group); ^£^ DHI severity (Level 1 reference group); items in bold with the sign * denotes items with *p* < 0.05 and effect size (DIF contrast) > 0.5.

**Table 7:** Standardised residual variance.

	All 25 Items			Physical Scale			Function Scale			Emotional Scale		
Variance	Eigenvalue	Observed (%)	Expected (%)	Eigenvalue	Observed (%)	Expected (%)	Eigenvalue	Observed (%)	Expected (%)	Eigenvalue	Observed (%)	Expected (%)
Total raw variance in observations	43.80	100	100	14.70	100	100	19.65	100.0	100.0	12.66	100.0	100.0
Raw variance explained by measures	18.80	42.9	42.7	5.70	38.8	38.4	10.65	54.2	54.2	5.66	44.7	43.9
Raw variance explained by persons	9.05	20.7	20.6	2.72	18.5	18.3	5.53	28.2	28.2	3.02	23.8	23.4
Raw variance explained by items	9.75	22.3	22.2	2.98	20.3	20.1	5.12	26.0	26.0	2.64	20.9	20.5

**Table 8:** Standardised residual variance.

	Unexplained Variance	Raw Unexplained Variance (Total)	1st Contrast	2nd Contrast	3rd Contrast	4th Contrast	5th Contrast
**Full scale**	Eigenvalue	25.0	3.4	2.1	1.8	1.6	1.3
	Observed (%)	57.1	7.8	4.8	4.1	3.7	3.0
	Expected	57.3	13.7	8.4	7.2	6.4	5.2
**Physical subscale**	Eigenvalue	9.0	1.7	1.4	1.3	1.1	1.0
	Observed (%)	61.2	11.7	9.6	9.0	7.6	7.0
	Expected (%)	61.6	19.1	15.6	14.7	12.4	11.4
**Function subscale**	Eigenvalue	9.0	1.9	1.5	1.2	1.0	1.0
	Observed (%)	45.8	9.4	7.4	6.2	5.3	5.2
	Expected (%)	45.8	20.6	16.2	13.6	11.5	11.4
**Emotional subscale**	Eigenvalue	7.0	1.7	1.2	1.2	1.1	1.0
	Observed (%)	55.3	13.6	9.7	9.1	8.5	7.9
	Expected (%)	56.1	24.6	17.6	16.4	15.4	14.3

## Data Availability

Data are not available upon request due to ethical restrictions.
